# m6A regulator–mediated RNA methylation modification patterns and immune microenvironment infiltration characterization in patients with intracranial aneurysms

**DOI:** 10.3389/fneur.2022.889141

**Published:** 2022-08-05

**Authors:** Aierpati Maimaiti, Mirzat Turhon, Xiaojiang Cheng, Riqing Su, Kaheerman Kadeer, Aximujiang Axier, Dilimulati Ailaiti, Yirizhati Aili, Rena Abudusalamu, Ajimu Kuerban, Zengliang Wang, Maimaitili Aisha

**Affiliations:** ^1^Department of Neurosurgery, Neurosurgery Centre, The First Affiliated Hospital of Xinjiang Medical University, Urumqi, China; ^2^Department of Neurointerventional Surgery, Beijing Neurosurgical Institute, Capital Medical University, Beijing, China; ^3^Department of Neurointerventional Surgery, Beijing Tiantan Hospital, Capital Medical University, Beijing, China; ^4^Department of Neurology, Neurology Centre, The First Affiliated Hospital of Xinjiang Medical University, Urumqi, China; ^5^Department of Neurosurgery, The First People's Hospital of Kashgar Prefecture, Kashgar, China

**Keywords:** intracranial aneurysm, epigenetics, m6A RNA methylation, immune microenvironment, immunity

## Abstract

**Background:**

The role of epigenetic modulation in immunity is receiving increased recognition—particularly in the context of RNA N6-methyladenosine (m6A) modifications. Nevertheless, it is still uncertain whether m6A methylation plays a role in the onset and progression of intracranial aneurysms (IAs). This study aimed to establish the function of m6A RNA methylation in IA, as well as its correlation with the immunological microenvironment.

**Methods:**

Our study included a total of 97 samples (64 IA, 33 normal) in the training set and 60 samples (44 IA, 16 normal) in the validation set to systematically assess the pattern of RNA modifications mediated by 22 m6A regulators. The effects of m6A modifications on immune microenvironment features, i.e., immune response gene sets, human leukocyte antigen (HLA) genes, and infiltrating immune cells were explored. We employed Lasso, machine learning, and logistic regression for the purpose of identifying an m6A regulator gene signature of IA with external data validation. For the unsupervised clustering analysis of m6A modification patterns in IA, consensus clustering methods were employed. Enrichment analysis was used to assess immune response activity along with other functional pathways. The identification of m6A methylation markers was identified based on a protein–protein interaction network and weighted gene co-expression network analysis.

**Results:**

We identified an m6A regulator signature of *IGFBP2, IGFBP1, IGF2BP2, YTHDF3, ALKBH5, RBM15B, LRPPRC*, and *ELAVL1*, which could easily distinguish individuals with IA from healthy individuals. Unsupervised clustering revealed three m6A modification patterns. Gene enrichment analysis illustrated that the tight junction, p53 pathway, and NOTCH signaling pathway varied significantly in m6A modifier patterns. In addition, the three m6A modification patterns showed significant differences in m6A regulator expression, immune microenvironment, and bio-functional pathways. Furthermore, macrophages, activated T cells, and other immune cells were strongly correlated with m6A regulators. Eight m6A indicators were discovered—each with a statistically significant correlation with IA—suggesting their potential as prognostic biological markers.

**Conclusion:**

Our study demonstrates that m6A RNA methylation and the immunological microenvironment are both intricately correlated with the onset and progression of IA. The novel insight into patterns of m6A modification offers a foundation for the development of innovative treatment approaches for IA.

## Graphical Abstract

Flowchart of this study. 
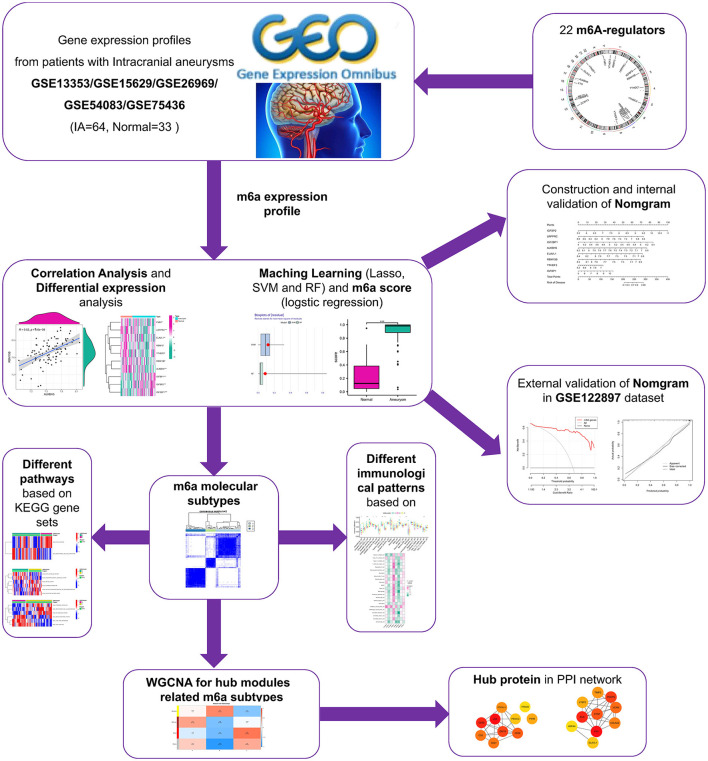

## Introduction

Intracranial aneurysm (IA) is a major contributor to non-traumatic subarachnoid hemorrhage, which poses a significant burden on global health (1). It is associated with a destructive central nervous system, a high rate of in-hospital mortality (45%) (2, 3), disability (30%), and incidence of long-term cognitive impairment (50%) among survivors (4, 5). According to previous studies, unruptured IA may exist in ~3% of the US population (6, 7) and 6–7% of the Chinese population (8). Available treatment modalities for IA include surgical clamping and various endovascular treatment options (e.g., endovascular coils, bypasses, and endovascular devices) (9). However, the choice of treatment modality and the timing of treatment initiation for IA in different conditions (ruptured or unruptured) remains controversial (10). In addition, the main treatments are invasive procedures that may cause a variety of complications. Therefore, effective management of unruptured IA (UIA) and treatment of IA remains a major clinical challenge. Improving our comprehension of the pathogenesis of IA is thus imperative to facilitate effective treatment strategies for this disease.

Previous research reports have demonstrated that inflammatory and immunological responses, hemodynamic stress, and extracellular matrix disintegration play a role in the etiology and rupture of IA (11, 12). Inflammation is emerging as a key component of IA pathophysiology (13). A high degree of inflammatory cell infiltration, including mast cells, is associated with human aneurysm rupture (14). Therefore, immunomodulation of aneurysm rupture prevention and IAs by modulating inflammation is considered a potential treatment for UIAs (15). Despite numerous investigations to clarify the molecular processes underlying IA rupture, only a few have performed gene expression profiling of the vessel wall tissue and post-clamping aneurysms in patients with IA to determine possible targets underlying the IA rupture mechanism. Hence, there is a need to find new biomarkers for the early clinical prognosis of patients with IA and to explore potential mechanisms of IA progression to develop novel treatment methods.

IA exhibits extensive genetic and phenotypic heterogeneity, characterized by epigenetic alterations. Epigenetics includes DNA methylation and reversible modifications of proteins (histones), such as acetylation, which can independently regulate gene expression outside of the DNA sequence (16). DNA methylation can contribute to heterochromatin formation and gene silencing, while histone acetylation is commonly thought to relax the chromatin structure and thus promote gene transcription (17). The functions of histone methylation are more diverse, with both transcriptional activation and repression (18). Conventionally, RNA modification was thought to represent the third layer of epigenetics, controlling RNA metabolism and processing (19). RNA alterations are present in all living organisms, and over 150 different modifications have been discovered, including N1-methyladenosine (m1A), N6-methyladenosine (m6A), m7G (20), 2'-oxo-methylation, 5-methylcytosine (m5C), and ac4C RNA acetylation (21). However, the internal alteration of RNA (known as m6A) is thought to be the most conserved, frequent, and abundant form (22). Ever since the discovery of RNA demethylases and the institution of sequencing protocols for methylated RNA, it has been recognized that the regulation of RNA processing, transcription, splicing variation, translation, and stability depend on methylation (23). The m6A modification occurs mainly on the adenine in the RRACH sequence and is determined by the “Writer,” “Eraser,” and “Reader” complexes. The encoder complex (Writer) is the methyltransferase comprising METTL3, WTAP, ZC3H13, RBM15, RBM15B, and CBLL1; while ALKBH5 and FTO act as demethylases (Eraser) to reverse methylation; and m6A recognized by m6A-binding proteins. m6A-binding proteins (Readers) are currently identified as *YTH* structural domain proteins *(YTHDC1, YTHDC2, YTHDF1, YTHDF2*, and *YTHDF3), HNRNP* family of nuclear inhomogeneous proteins *(HNRNPC)*, as well as *ELAVL1, IGFBP1, IGFBP2, IGFBP3, HNRNPA2B1, LRPPRC, FMR1*, and *IGF2BP1* (24).

The encoder modulates the accumulation of the m6A function, whereas the decoder modulates its depletion (25). Encoders and decoders are essential for the maintenance of a dynamic equilibrium of the levels of m6A in cells and tissues. Post-transcriptional gene expression may be subjected to certain influence by readers (m6A-binding proteins) in response to the accumulation of m6A on natural RNA transcripts upon transcription (26). It has recently been suggested that the modulation of the *m6A* gene can be used to elucidate the underlying mechanism of immunological regulation. Depleted *METTL3* expression attenuates the degrading of RIPK2 and NOD1 mRNA *via* the actions of YTHDF1 and YTHDF2, which upmodulates the NOD1 pathway thereby increasing the lipopolysaccharide-elicited inflammatory process in macrophages (27). It has also been shown that METTL3-mediated m6A modification ensures antiviral immunity by promoting mRNA stability and protein translation (28). In addition, Zhou et al. showed that YTHDC1 deficiency leads to M1 microglia polarization, increased inflammatory response, and promotes microglial migration. Mechanistically, YTHDC1 maintains the stability of sirtuin 1 (SIRT1) mRNA, which reduces the phosphorylation of signal transducer and activator of transcription 3 (STAT3), and is crucial for the regulation of microglial inflammatory responses (29). Despite growing evidence for the regulatory role of m6A in immune response, no research has focused on the role of m6A in the pathogenesis of IA. Therefore, analyzing the immune alterations between normal tissue (superficial temporal arteries) and IA samples, as well as between different subtypes of IAs and alterations in m6A modulator levels could provide unique insight into the pathogenesis of IAs.

In this study, we investigated the patterns of m6A modulator modifications in IAs in a systematic manner. We discovered that m6A modulators could well differentiate between normal tissue and IA samples. A significant correlation was observed between the infiltrating immune cell abundance and immune response gene sets in IAs and m6A modulators, signifying a close-fitting binding between immune modulators and m6A modulators. We aggregated IA samples according to 22 m6A modulators and identified three distinct m6A modification patterns. Distinct immunological profiles were detected in different isoforms, and we performed a comparison of the biological roles of these isoforms. These aforementioned studies suggest that m6A modification patterns have a remarkable impact on the immunological microenvironment of IAs.

## Materials and methods

### Data source and pre-processing of IA

We downloaded the following RNA-seq datasets from the Gene Expression Omnibus (GEO) database (https://www.ncbi.nlm.nih.gov/gds/): GSE13353 (30), GSE15629 (31), GSE26969 (32), GSE54083 (33), and GSE75436. These five gene sets were used as the screening set, and the “sva” package of R x64.4.0.3 was employed to de-batch the raw data (Supplementary Figure S1) that included a total of 64 cases of IA samples and 33 of normal samples. In addition, the GSE122897 (34) dataset was downloaded as the validation set, which included 44 cases of IA samples and 16 cases of normal samples. The above samples were taken from the same tissue type, and detailed clinical features of patients and platform files are available in Supplementary File 1. In addition, 22 m6A modulators were annotated in the final normalized dataset according to the inclusion of m6A-associated regulators from previous literature. These included *ALKBH5, ZC3H13, IGF2BP1, RBM15 RBM15B, HNRNPA2B1, CBLL1, LRPPRC, FMR1, HNRNPC, IGFBP1, IGFBP2, IGFBP3, YTHDC1, YTHDC2, YTHDF1, YTHDF2, YTHDF3, ELAVL1, WTAP, FTO*, and *METTL3*.

### Differences in m6A regulators between different samples and correlation analysis

The Wilcox test was performed for the purpose of assessing differences in the expression level of m6A modulators between normal and IA samples. Expression relationships between m6A modulators were assessed by Spearman correlation analysis in both whole and IA samples, focusing on the correlation between erasers and writers.

### Screening of core m6A regulators

We adopted the LASSO regression (10-fold) method to remove redundant genes from 22 regulators; based on the removal of redundant genes, both support vector machine (SVM) and random forest (RF) models were constructed, and the residuals were calculated to compare the advantages and disadvantages of the two models. RF is a component-supervised learning technique that might be regarded as an extension of decision trees. The structural risk minimization concept of statistical learning theory underlies the SVM method, which is a kind of supervised machine learning algorithm. Following plotting each data point as a point in an *n*-dimensional space (with *n* indicating the number of m6A modulators), an optimal hyperplane is determined that can distinguish between the two classes (normal and IA samples). After determining the optimal machine learning model, the m6A regulators associated with the occurrence of IA were identified by one-way logistic regression with a threshold of *P* < 0.05. The corresponding coefficients for each interval of m6A regulators were subsequently calculated by multi-factor logistic regression, before obtaining the final scores.

### Identification and evaluation of nomogram

The “rms” R package was used to plot column line plots to construct a nomogram. A calibration curve, area under the receiver operating characteristic (AUC of ROC) curve, clinical impact curve, and risk decision curve analysis (DCA) were used to assess the discriminatory performance of the scores.

### Determination of the m6A modification pattern

According to the expression levels of core m6A modulators, we utilized an unsupervised cluster analysis technique to determine various m6A modification patterns. To evaluate the number of clusters and robustness, the consensus clustering approach was employed. The *k*-means clustering method with 100 iterations (utilizing 80% of samples each time) was used to ensure cluster stability. The clustering score of the cumulative distribution function (CDF) curve was used to estimate the optimum number of clusters. The reliability of consensus clustering was verified by performing a PCA analysis.

### Differences in immune characteristics and correlation analysis

We conducted the single-sample gene set enrichment analysis (ssGSEA) to predict the number of specific infiltrating immune cells as well as the activity of specific immunological responses. Based on the gene sets, we explored the status of immune cells and immune-related pathways. By performing the Kruskal-Wallis test, we made comparisons of the enrichment scores of immune cells and immune-related pathways between normal and IA samples. With the use of Spearman's correlation analysis, we evaluated the correlation between core m6A modulators and human leukocyte antigen (HLA) expression, immune cells, and immune response activity. In addition, the same method was employed to compare the immunological differences among various m6A modification patterns.

### Analysis of the biological enrichment of various m6A modification patterns

The gene set “c2.cp.kegg.v7.4.symbols” downloaded from the MSigDB database was used to reflect changes in biological signaling pathways. The expression matrix was converted into a scoring matrix using the gene set variation analysis (GSVA) algorithm and the scores of biological signaling pathways were compared between different m6A patterns by the “limma” R package with a threshold of *P* < 0.05 for differential analysis.

### Determination of differentially expressed genes (DEGs) between different m6A patterns

The “limma” R package was employed for the purpose of identifying DEGs between different m6A patterns, with the screening criterion set at *P* < 0.05. Additionally, Gene Ontology (GO) and Kyoto Encyclopedia of Genes and Genomes (KEGG) enrichment analyses were performed with the aid of the “clusterProfiler” R package.

### Weighted gene co-expression network analysis (WGCNA)

Data from expression matrices composed of genes with m6A modification patterns mediating differences were subjected to an evaluation utilizing the “WGCNA” R package. A WGCNA network was constructed and unsigned topological overlap matrices were utilized to detect modules. The optimum soft threshold was 8, the least number of genes in the module was 20, and the module truncation height was 0.2. The correlation of the merged modules with different m6A modification patterns was calculated using the Spearman method. Finally, the core proteins within the module were defined as the top 10 genes ranked following the MCC method in the protein–protein interaction (PPI) network. Visualization was performed using Cytoscape v3.7.1.

## Results

### Expression landscape of m6A modulators among different samples

**Figure 1:**
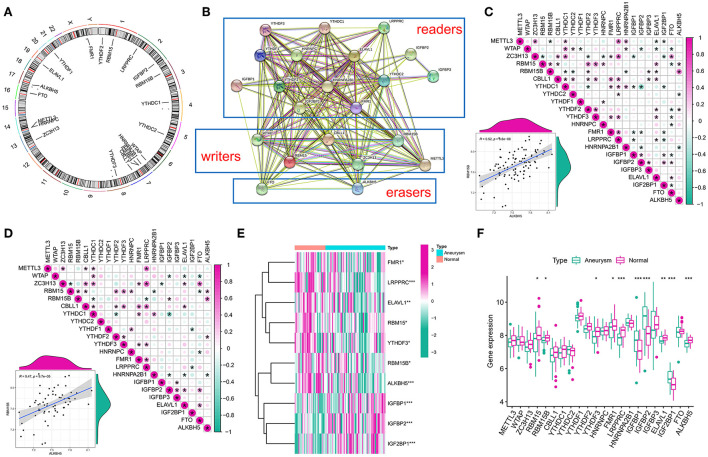
Landscape of gene and expression changes of m6A modulators in the intracranial aneurysm (IA). **(A)** Position of the CNV alteration of the m6A modulators on 22 chromosomes from the GSE13353, GSE15629, GSE26969, GSE54083, and GSE75436 cohorts. **(B)** Overview of the composition of m6A modulators as well as the protein–protein interactions (PPI) across 22 m6A RNA methylation modulators. **(C,D)** Relations between the expression levels of 22 m6A modulators in all samples and intracranial aneurysm samples. The two scatter plots indicate the two most correlated m6A modulators: ALKBH5 and RBM15B. **(E)** Heatmap of remarkable differences in the expression levels of 10 modulators in IA and normal samples. **(F)** Box plot of the transcriptional state of the expression levels of 22 m6A modulators in IA and normal samples. The symbols *, **, and *** indicates the value of *p* < 0.05, *p* < 0.01, and *p* < 0.001 respectively.

Twenty-two m6A modulators were included in the study, which included 2 erasers, 14 readers, and 6 writers. Figure 1A outlines the location of the m6A modulators on the chromosome. The regulatory interactions among these m6A modulators are expressed as a PPI network (Figure 1B) with the writers intricately correlated with each other and usually functioning as a complex. Subsequently, the correlated expression of different regulators was explored in the whole sample (Figure 1C) and in the IA sample (Figure 1D), where the focus was on the correlation between erasers and writers. A strong correlation was found between *ALKBH5* and *RBM15B* in each sample (*r* = 0.52, *r* = 0.47). In addition, the Wilcox test showed remarkable differences in the expression levels of 10 modulators in different samples (Figures 1E,F), such as *RBM15, RBM15B, YTHDF3, FMR1, LRPPRC, IGFBP1, IGFBP2, ELAVL1, IGF2BP1*, and *ALKBH5*.

### m6A regulators as potential biomarkers of IA

**Figure 2:**
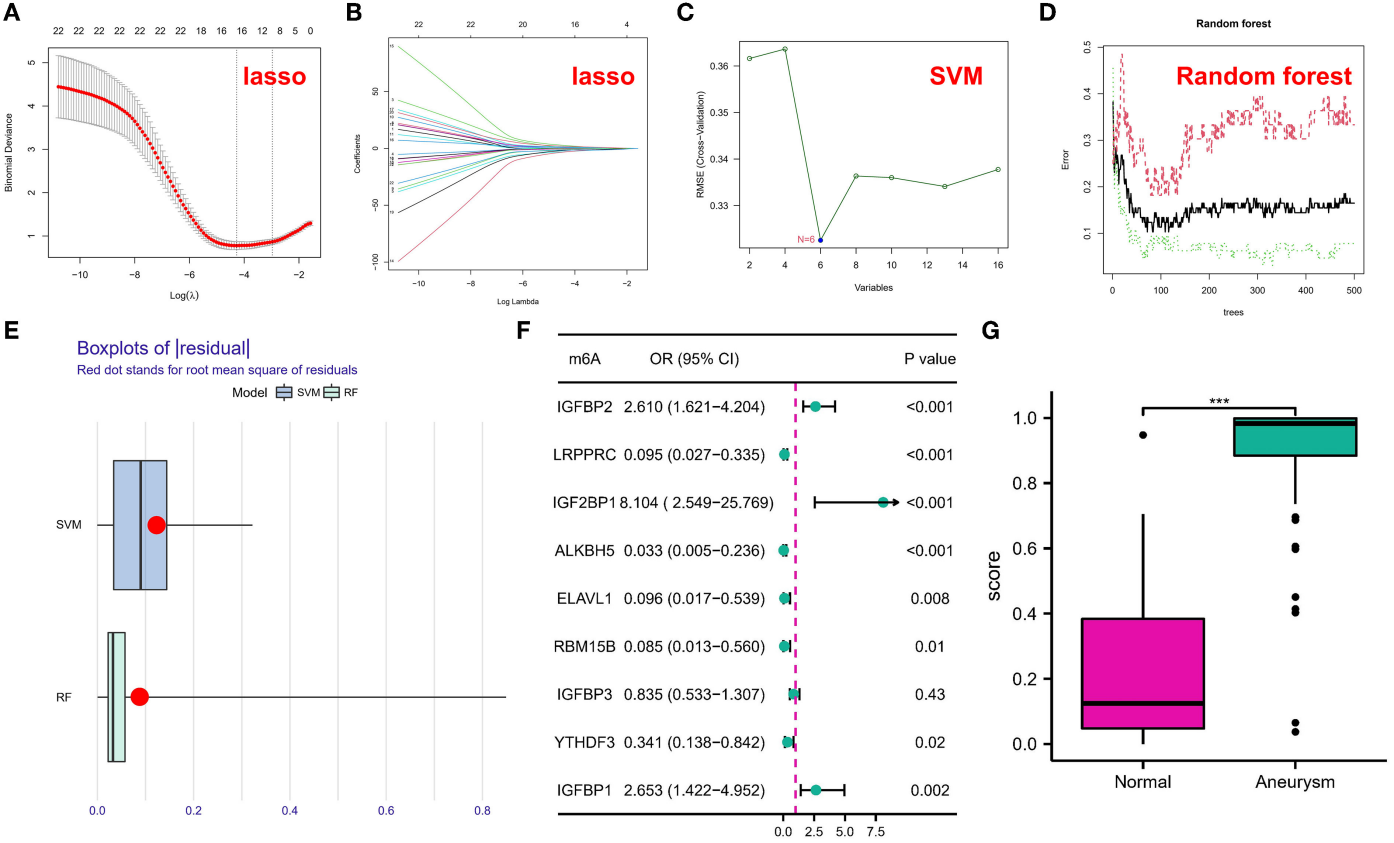
**(A,B)** Coefficient profiles of intracranial aneurysm (IA)-associated m6A modulators using the least absolute shrinkage and selection operator (Lasso). Cross-validation by 10-fold regression is used to fine-tune the parameter selection of Lasso. **(C)** Support vector machine (SVM) models. **(D)** Error graph of the random forest (RF) models. **(E)** Box plots of residuals in SVM and RF. **(F)** Correlation between m6A regulators and IA was explored utilizing univariate logistic regression, identifying nine m6A modulators associated with IA (*P* < 0.05). **(G)** Risk distribution for IAs and normal samples, with IAs having a significantly elevated risk score as opposed to normal samples. The symbol *** indicates he value of *p* < 0.001.

All of our data were normalized and genes with zero expression in the sample (>90% samples) were removed before inclusion in the machine learning model. To investigate the contribution of m6A regulators to IA pathogenesis, we conducted Lasso regression on 22 regulators for dimensionality reduction and feature selection to exclude redundant genes (Figures 2A,B), and 16 genes were finally used for subsequent analysis. Subsequently, SVM (Figure 2C) and RF (Figure 2D) models were developed to identify candidate m6A modulators from the 18 modulators to anticipate the onset of the IA. The residual box line plot (Figure 2E) shows that the RF model had the smallest residuals. Therefore, the RF model was chosen as the best fit. Following the determination of the importance and order of genes, those with importance scores <2 (total 9) were selected for screening by a one-way logistic regression (Figure 2F). Considering it would be difficult to use the black box (such as deep learning, RF, etc.) alone for clinical applications, it is with this in mind that we put logistic regression after machine learning. Finally, significant genes were integrated into the multifactorial logistic regression model and the final coefficients were determined as follow: score = expression of *IGFBP2* × 1.4389568 + expression of *LRPPRC* × −2.6183281 + expression of *IGF2BP1* × 2.7552530 + expression of *ALKBH5* × −4.6602717 + expression of *ELAVL1* × −3.7870236 + expression of *RBM15B* × −3.9206072 + expression of *YTHDF3* × −0.9092704 + expression of IGFBP1 × 0.5572252. The classifier consisted of eight regulators, in which IA scores were much higher than normal samples (Figure 2G). The ROC curve showed that the classifier had good diagnostic performance in classifying normal and IA samples (AUC=0.954; Supplementary Figure S2A). The classifier also had good diagnostic performance in the independent validation set GSE122897 (AUC=0.884; Supplementary Figure S2B).

### Construction of nomogram model

**Figure 3:**
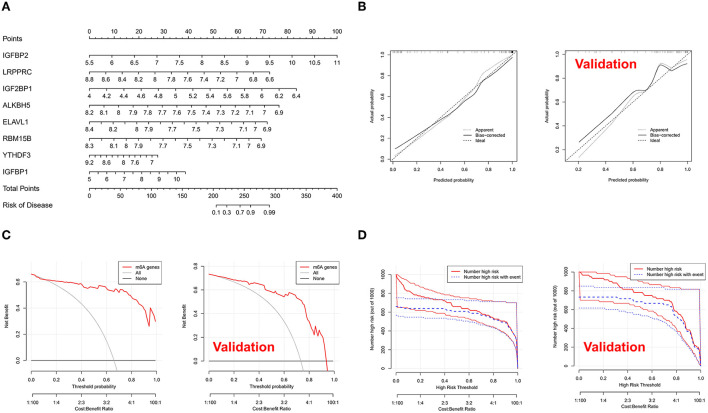
**(A)** Nomograms for estimating the risk scores of eight m6A modulators associated with intracranial aneurysm (IA). Each parameter was assigned a score, and the sum was transformed to the least possible probability. **(B)** Calibration plots of the model concerning the agreement between anticipated and actual IA in the training and testing cohorts. On the *X*-axis, the nomogram-anticipated IA is displayed, whereas the actual IA is presented on the *Y*-axis. The diagonally dotted line denotes flawless calibration using an ideal model that exactly represents the observed outcomes. The solid line denotes the real performance of the nomogram; a tight alignment of the dotted and solid lines indicates a more accurate assessment of the true outcomes. Decision curves for two IA-specific risk predictive models **(C)**. On the vertical axis, the net advantage of standardization is depicted. The correlation between the risk criterion and the cost-benefit ratio is represented by the two horizontal axes. The clinical impact curves of the models are displayed in **(D)**.

Utilizing eight m6A modulators as building blocks, a nomogram was constructed (Figure 3A). The calibration curves for both the training set and the independent validation set illustrated that the predictions of the column line graph model were correct (Figure 3B). Patients with IAs may gain more benefit from choices made based on the column line graph model, as evidenced by the fact that the red line in the DCA curve remained above the gray line (Figure 3C). The clinical impact curve showed significant predictive performance of the column line graph model (Figure 3D).

### m6A modulators are linked to immune responses in IA

**Figure 4:**
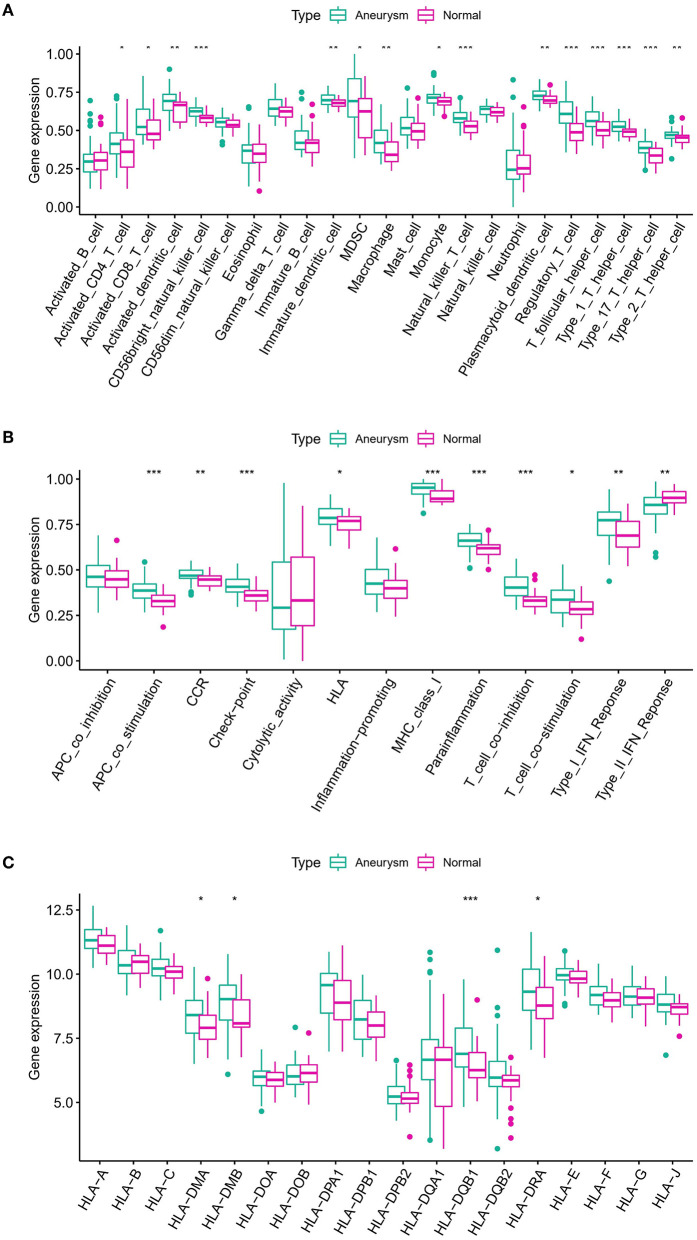
**(A)** Differences in the abundance of 23 infiltrating immunocytes. **(B)** Differences in the activity of 13 immune response gene sets in IA and normal subtypes. **(C)** Differences in the expression of 18 HLA genes between IA and normal subsets. The symbols *, **, and *** indicates the value of *p* < 0.05, *p* < 0.01, and *p* < 0.001 respectively.

**Figure 5:**
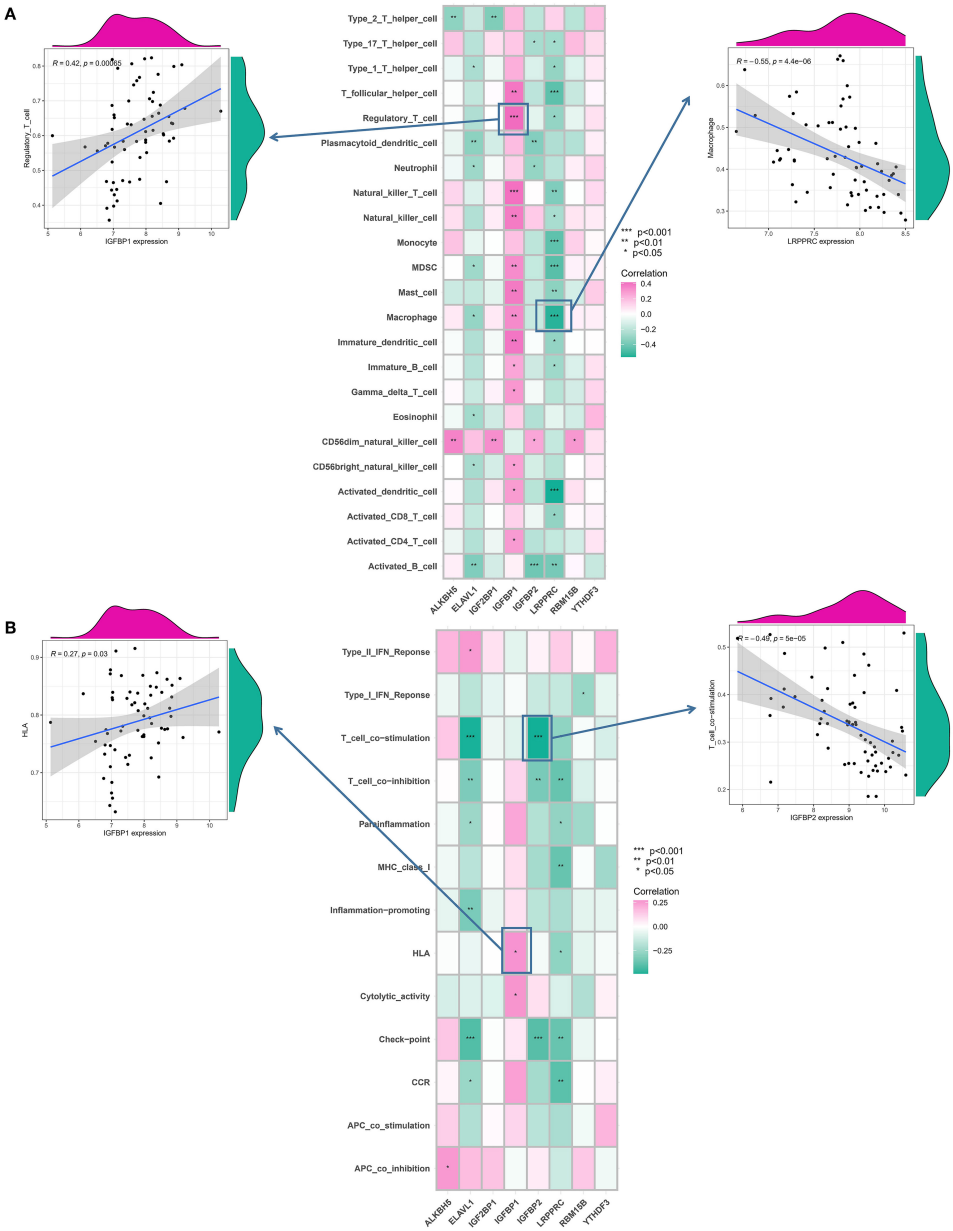
Correlation between immunocytes infiltration, immunological response gene-sets, and m6A modulators. **(A)** Correlation between each dysregulated m6A modulator and dysregulated immunological microenvironment infiltration cell type is illustrated by the square plot. The IGFBP1-regulatory T-cell pair has the highest positive correlation between immunocyte and m6A modulator. LRPPRC–macrophage pair has the most negative correlation between immunocyte and m6A modulator. **(B)** Correlation between each dysregulated m6A modulator and dysregulated immunological response gene set using a square plot. Intracranial aneurysms (IAs) had the highest positive correlation between IGFBP1 and HLA, suggesting elevated expression level of IGFBP1 and highly active HLA in IA. IGFBP2–T cell co-stimulation pair has the strongest negative correlation. The symbols *, **, and *** indicates the value of *p* < 0.05, *p* < 0.01, and *p* < 0.001 respectively.

To examine the biological behavior between the immunological microenvironment and m6A modulators, we correlated the expression of the above eight core regulators with infiltrating immune cells and immune-related pathways. Differential analysis revealed differences between healthy samples and IA samples in the abundance of infiltrating cells in the immune microenvironment, immune function, and HLA expression, with most of the natural killer immune cells altered in IA samples relative to the normal samples, including macrophages, activated T cells, etc. (Figure 4A). In addition, significant activation of TYPE 1 inflammatory response pathway was observed in IA samples, whereas a significant activation of TYPE 2 inflammatory response pathway was observed in normal samples, suggesting that this pathway is involved in the inflammatory process (Figure 4B). HLA-DRA, HLA-DQB1, HLA-DMB, and HLA-DMA were also significantly upregulated in IA samples (Figure 4C). Correlation analysis revealed that eight co-regulators were closely correlated with a variety of immune cells in IA samples (Figure 5A). For example, the abundance of LRPPRC macrophages had the strongest negative correlation (*r* = −0.55) and IGFBP1 demonstrated the strongest positive correlation with regulatory T cell abundance (*r* = 0.42). In terms of immune function, we found that insulin-like growth factor binding protein 2 (IGFBP2) exhibited the strongest negative correlation with T-cell costimulatory pathway (*r* = −0.49) and IGFBP1 exhibited the strongest positive correlation with HLA (*r* = 0.27) (Figure 5B). In addition, LRPPRC exhibited the strongest positive and negative correlation with ALKBH5 and HLA-G, respectively (Supplementary Figure S3). The above results demonstrate that the core m6A modulator performs a fundamental role in the IA immunological microenvironment.

### Modification patterns mediated by m6A modulators in IA

**Figure 6:**
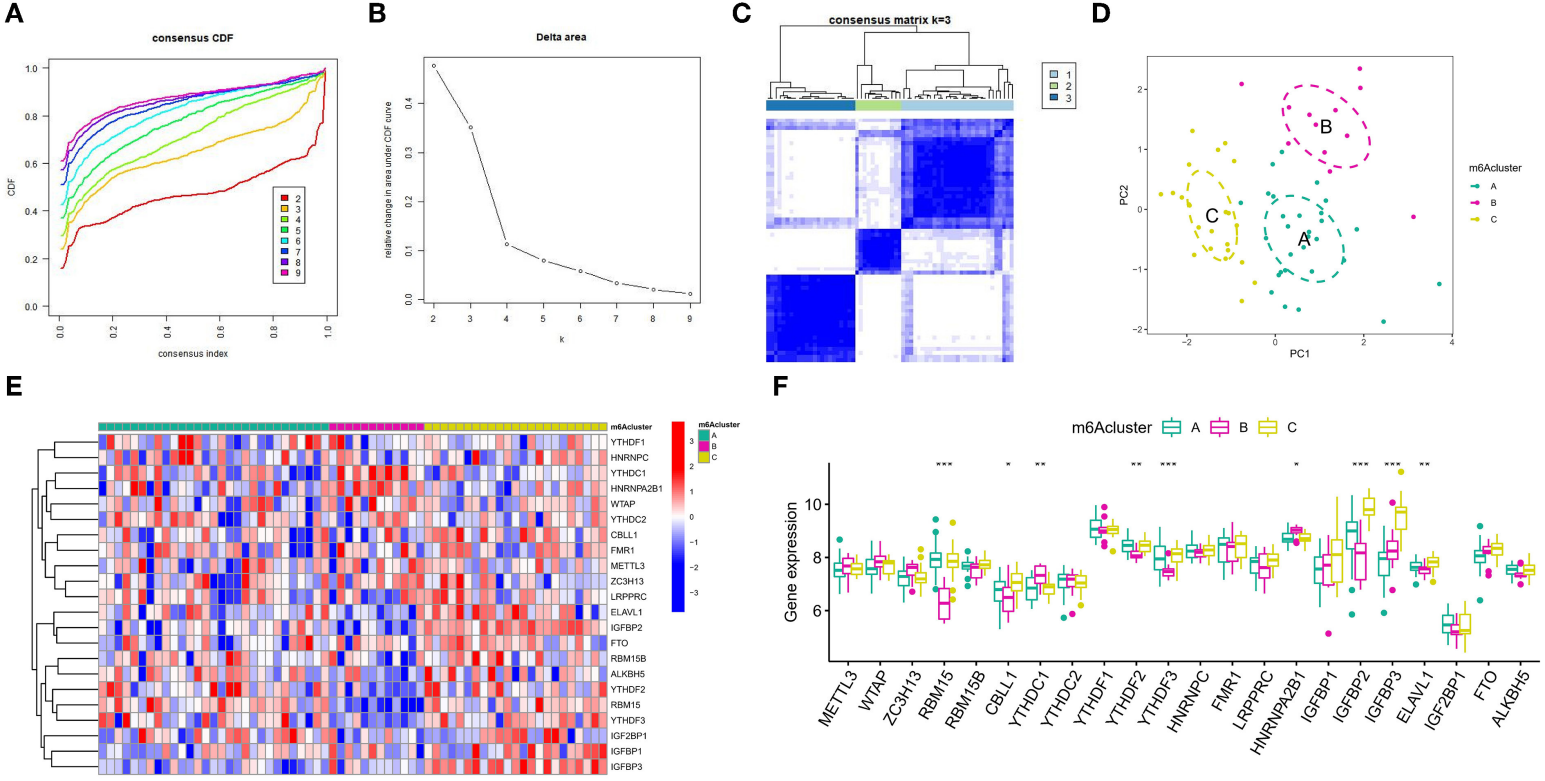
Unsupervised clustering of 22 distinct m6A modulators. Three different m6A modification patterns in the intracranial aneurysm. **(A)** Cumulative distribution function (CDF) for consensus clustering for *k* = 2–9. **(B)** Relative change in the area under the CDF curve for *k*=2–9. **(C)** Matrix of co-occurrence percentages for cerebral aneurysm samples visualized as a heatmap. **(D)** Principal component analysis was used to analyze the transcriptome profiles of three m6A subtypes. A striking divergence in the transcriptome across the various modification patterns can be seen. **(E)** Unsupervised clustering of 22 m6A regulators in the three modification patterns. **(F)** State of expression of 22 distinct m6A modulators in each of the three m6A subsets. The symbols *, **, and *** indicates the value of *p* < 0.05, *p* < 0.01, and *p* < 0.001 respectively.

According to the expression levels of modulators, we conducted an unsupervised consistent clustering analysis of 63 IA samples (Figures 6A–C). We identified three different m6A modification isoforms, and PCA analysis showed that patients with IA could be classified into three clusters according to m6A modulators (Figure 6D). The expression of some m6A modulators was significantly different among different modification patterns (Figures 6E,F).

### Immune microenvironment and biological functional characteristics in distinct m6A modification patterns

**Figure 7:**
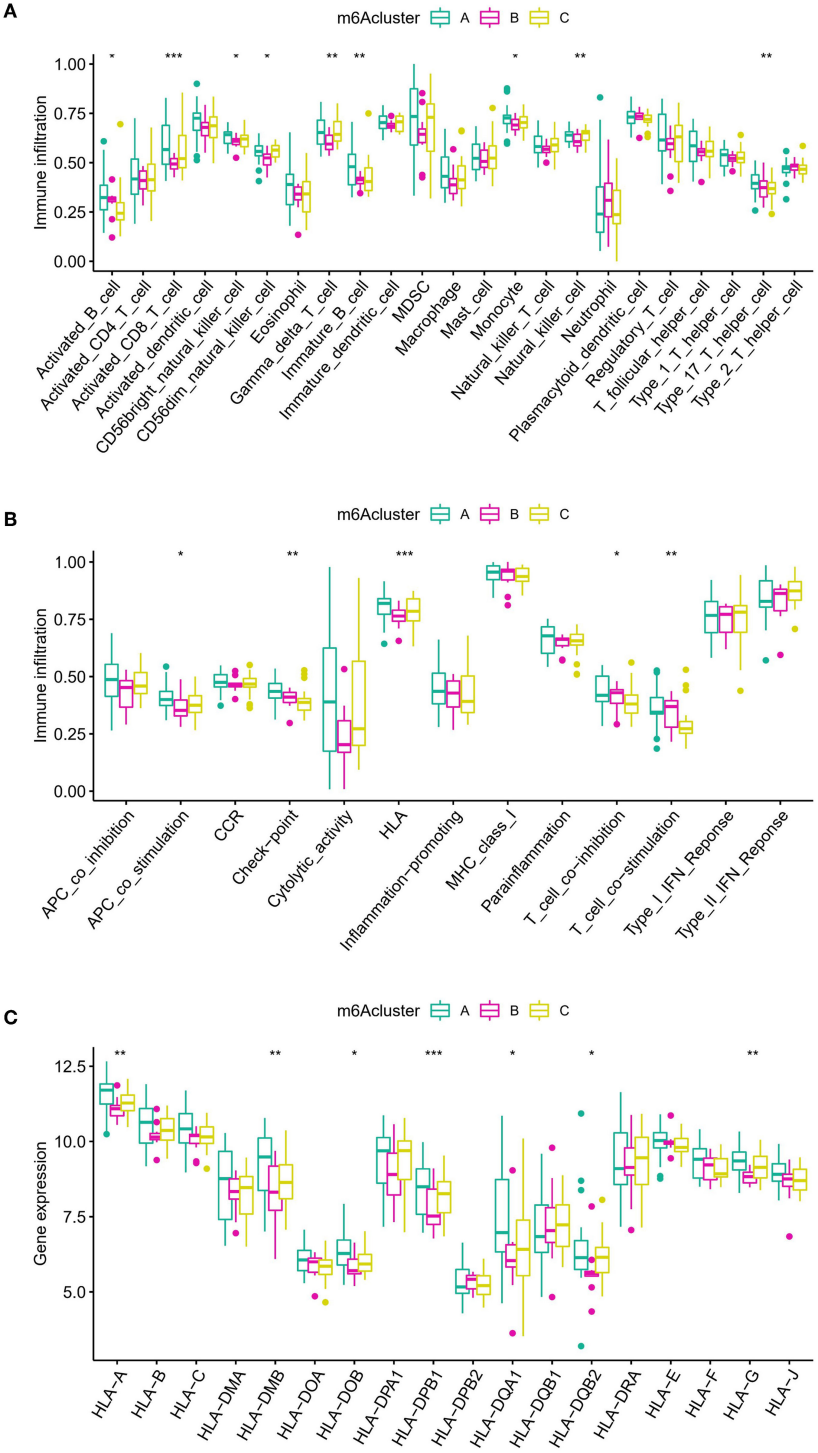
Diverse aspects of the immunological microenvironment associated with various m6A modification patterns. **(A)** Differences in the abundance of each immunological microenvironment infiltrating immunocyte in three different m6A modification patterns. **(B)** Differences in the activity between each immune response gene in three m6A modification patterns. **(C)** Differences in the expression level of each HLA gene across three m6A modification patterns. The symbols *, **, and *** indicates the value of *p* < 0.05, *p* < 0.01, and *p* < 0.001 respectively.

To determine the variations in immunological microenvironmental characteristics between these distinct m6A modification patterns, differences between infiltrating immune cells and immune function were assessed. Compared with patterns B and C, pattern A had higher activated T cells and activated natural killer cells (Figure 7A). Concerning the immune response, pattern A had a more active immune response (Figure 7B). In addition, the expression of different HLAs also differed between the modification patterns (Figure 7C). The above findings additionally demonstrate that m6A modification plays an important modulatory role in the formation of different immunological microenvironments in IA.

### Biological properties of different m6A modification patterns

**Figure 8:**
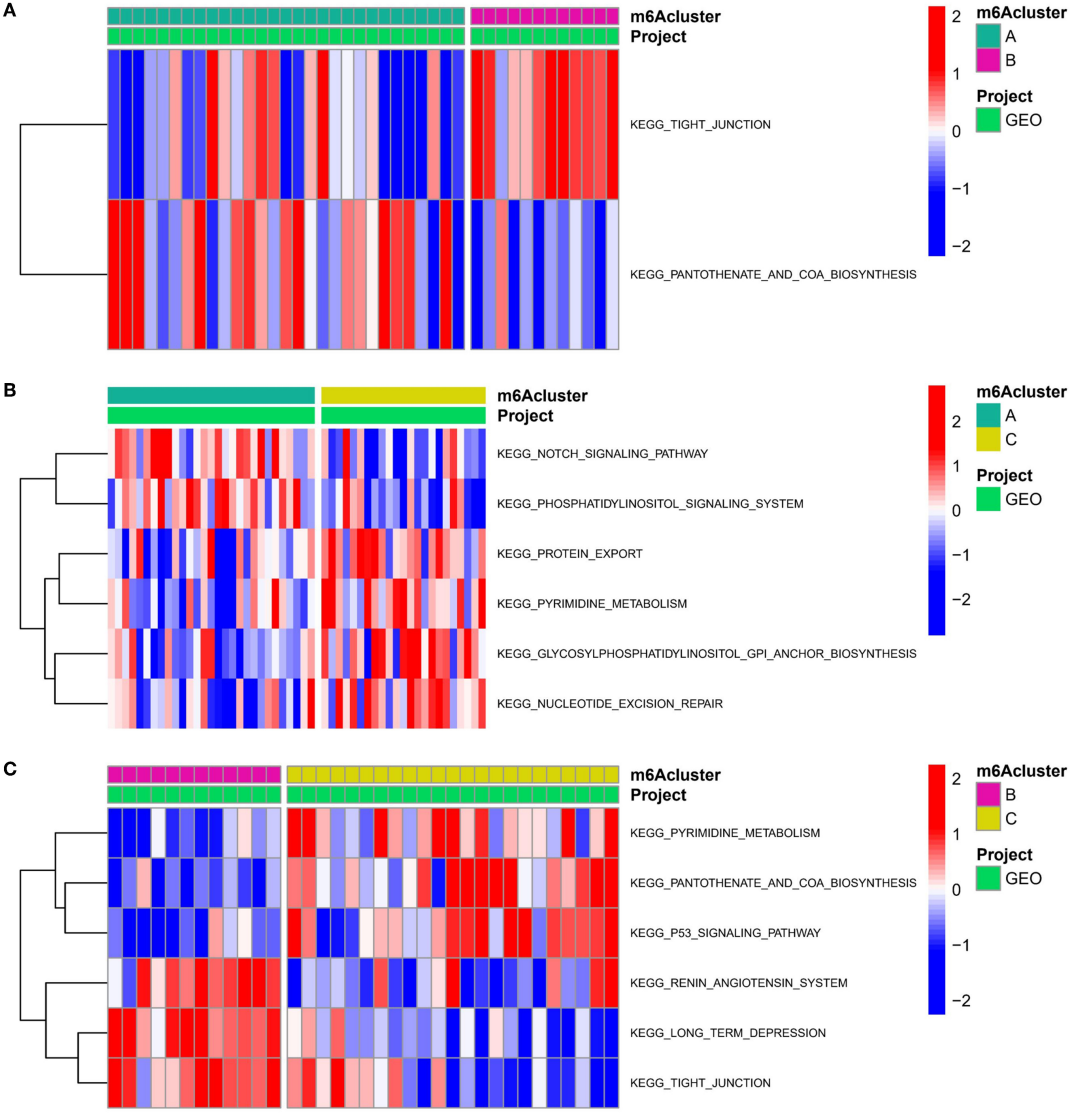
Underpinned biological functional features of three m6A modification sequences. **(A–C)** Differences in the KEGG pathway enrichment scores for m6A modification **(A)** patterns A and B; **(B)** patterns A and C; and **(C)** patterns B and C.

**Figure 9:**
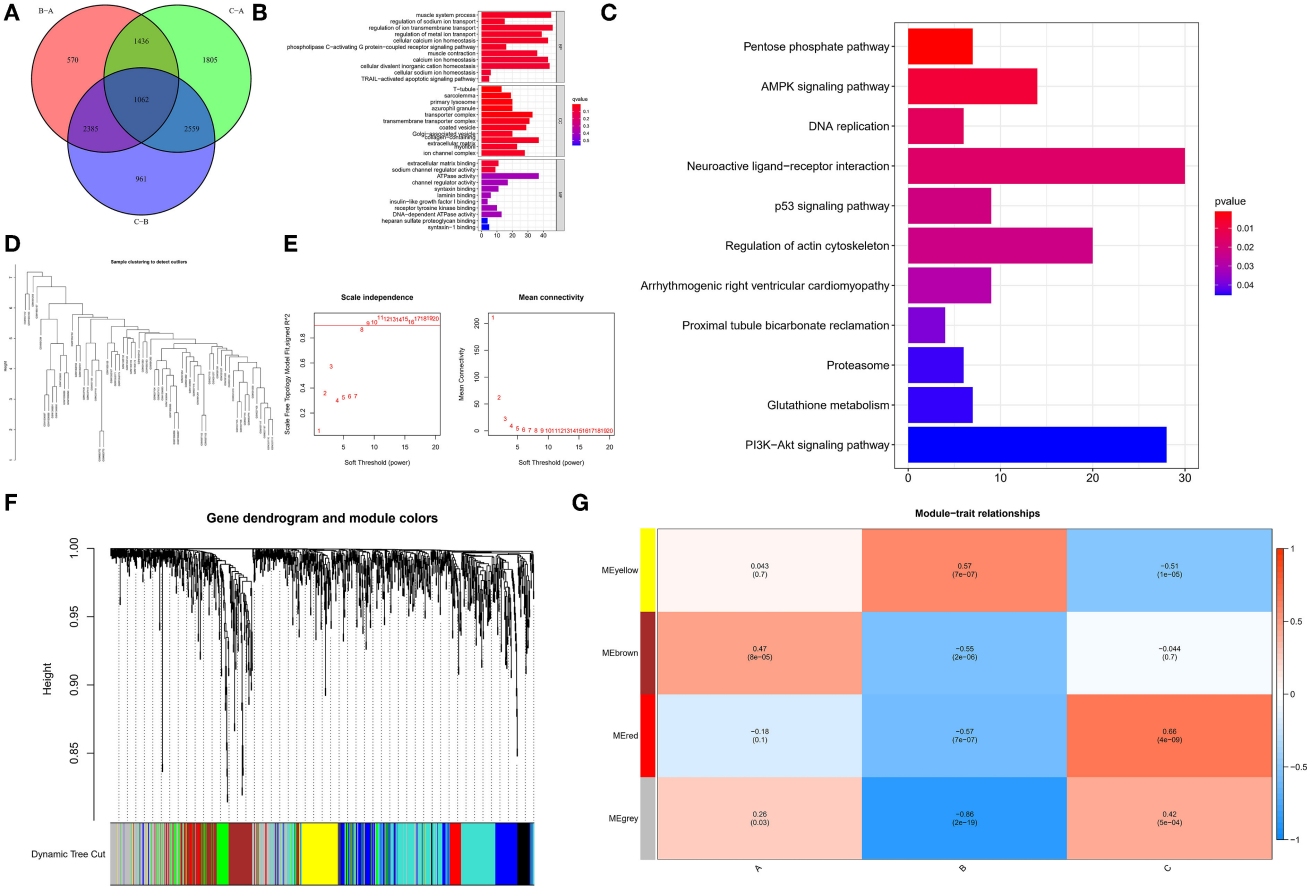
Determination and functional analysis of genes associated with the m6A phenotype in IA. **(A)** Venn diagram of 1,062 genes associated with the m6A phenotype. **(B)** Assessment of GO-BP, CC, and MF functional enrichment of the biological features of genes associated with the m6A phenotype. **(C)** KEGG enrichment analysis of immune genes associated with the m6A phenotype to elucidate the correlation between m6A modulators and immunological modulation. **(D)** Clustering of samples according to the expression data from all samples. WGCNA analyzed the top 25% of variation genes, excluding outlier data. **(E)** The scale-free ft-index, as well as the mean connectivity, were analyzed for a range of soft-thresholding powers. **(F)** Gene dendrogram generated using hierarchical clustering based on average linkage. The color row below the dendrogram indicates the module allocation established by the Dynamic Tree Cut, which identified four modules. **(G)** Heatmap of the correlation between module eigengenes and m6A modification patterns.

To examine the biological responses in the three m6A modification patterns, we compared their respective KEGG pathways and performed a GSVA enrichment analysis to investigate the status of biological pathway activation. The tight junction was significantly enriched in model A compared to model B (Figure 8A). The Notch signaling pathway was significantly enriched in model A compared to model C (Figure 8B). The p53 signaling pathway was significantly enriched in pattern C compared to pattern B (Figure 8C). In addition, a total of 1,062 DEGs with different modification patterns were identified (Figure 9A; Supplementary File 2), and subsequent GO enrichment analysis revealed their involvement in processes, such as muscular system processes, regulation of ion transport, various cation homeostasis, and TRAIL-activated apoptotic signaling pathways (Figure 9B). These results were consistent with IAs in which dysfunction is known, indicating the reliability of our results. In addition, in the KEGG analysis, the screened DEGs were significantly associated with the pentose phosphate pathway, AMPK signaling pathway, DNA replication, neuroactive ligand–receptor interaction, p53 signaling pathway, regulation of actin cytoskeleton, arrhythmogenic right ventricular cardiomyopathy, and other pathways (Figure 9C). We further identified gene–gene modules associated with different m6A modifications in the 1,062 DEGs using the WGCNA method (Figures 9D–F). A total of four gene modules were identified with distinct modification patterns matching their associated genes (Figure 9G), with brown modules linked to subtype A (*r* = 0.47), gray modules linked to subtype B (*r* = −0.86), and red modules linked to subtype C (*r* = 0.66).

### Characterization of coregulatory proteins with different m6A modification patterns

**Figure 10:**
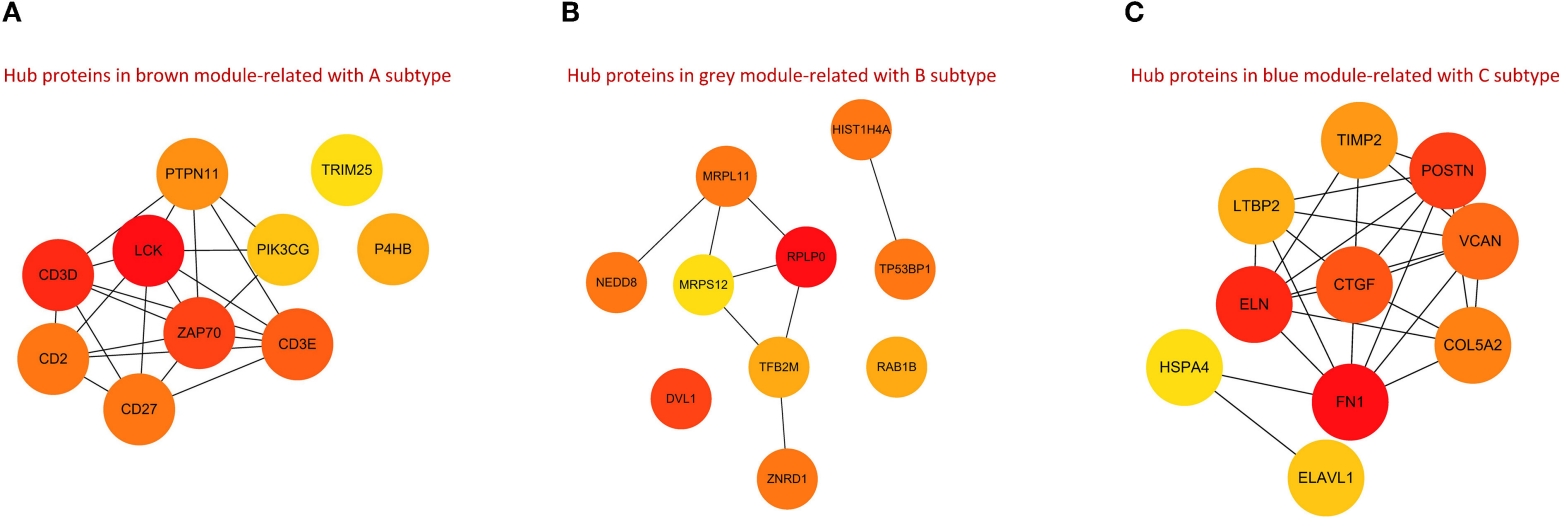
PPI networks for hub proteins in brown module-related to A subtype **(A)**, gray module-related to B subtype **(B)**, and blue module-related to C subtype **(C)**.

The ppi networks of the brown, gray, and red modules were constructed in the STRING database, and the MCC values of each protein were calculated in Cytoscape. Among them, we found that subtype A may be primarily regulated by LCK, CD3D, CD2, CD27, CD3E, ZAP70, PTPN11, PIK3CG, TRIM25, and P4HB (Figure 10A); subtype B by HIST1H4A, TP53BP1, RAB1B, MRPL11, RPLP0, MRPS12, TFB2M, ZNRD1, NEDD8, and DVL1 (Figure 10B); and subtype C by FN1, ELN, CTGF, LTBP2, COL5A2, VCAN, POSTN, TIMP2, HSPA4, and ELAVL1 (Figure 10C).

## Discussion

Epigenetic alterations in DNA have been extensively investigated in many diseases, contributing to the development of various treatment approaches, such as histone deacetylase inhibitors, DNA methyltransferases, and immunomodulatory therapies (35, 36). m6A is the most common form of mRNA modifications in eukaryotes and functionally regulates the eukaryotic transcriptome (37), which affects mRNA splicing, export, localization, translation, and stability (38). There has been a large amount of evidence to support the view that m6A methylation modifications perform an instrumental role in the onset and progression of a variety of illnesses, including malignant tumors (39). Nevertheless, there is a scarcity of studies on m6A methylation in IA. Our research was the first to probe into the involvement of m6A modulators in IA with the aim to discover a correlation between m6A methylation modifications and immunological features. We observed that the expression of m6A modulators differed significantly between normal and IA tissues in nearly half of all tissues. We identified an m6A regulator gene signature (*IGFBP2, LRPPRC, IGF2BP1, ALKBH5, ELAVL1, RBM15B, YTHDF3, IGFBP1*) after using Lasso regression, machine learning (SVM and RF), and univariate and multivariate logistic regression. The IA and normal samples were easily distinguished based on the differences in m6A methylation modification patterns between them.

Of the 23 m6A regulator genes identified in the present research, the m6A regulator gene signature was the most crucial, due to its substantial fold-changes and significance in the multivariate analysis. In addition, numerous m6A regulators had protein interactions or expression correlations, demonstrating a regulatory network of m6A modifications. Second, we explored the correlation between m6A modulators and immunological features of IAs, which included infiltrating immune cells, HLA gene expression, and immune response gene sets. We discovered that most of the m6A modulators were intimately associated with these immunological features, suggesting an integral function of m6A modifications in the modulation of the immune microenvironment in IAs. Macrophage abundance exhibited the strongest negative correlation with LRPPRC, and regulatory T-cell abundance exhibited the strongest positive correlation with IGFBP1. In terms of immune function, IGFBP2 exhibited the strongest negative correlation with the T-cell co-stimulatory pathway and IGFBP1 exhibited the strongest positive correlation with HLAs. In addition, LRPPRC and ALKBH5 exhibited the strongest positive and negative correlations, respectively, with HLA-G, and played a crucial role in IA homeostasis.

Previous studies have shown that recombinant IGFBP1 and PYY primary human CD4 T cells are, respectively, characterized by their blocking and induction of immune activation (40). It was also shown that the inflammation-related cytokines IGFBP1 and RANTES diminished the megakaryocytic potential of hematopoietic stem cells after transplantation in patients with prolonged isolated thrombocytopenia. Among them, IGFBP1 was found to be regulated upon activation, and its expression and secretion by normal T cells significantly inhibited the proliferation of hematopoietic stem cells as well as the differentiation of megakaryocytes *in vitro* (41). In addition, patients with severe and moderate Alzheimer's disease demonstrated a progressive elevation in the expression levels of IGFBP1 protein in their blood profile (42). However, there were no relevant reports for LRPPRC and macrophages. We utilized m6A modulator expression profiles to conduct unsupervised clustering of IA samples. This resulted in the identification of three subtypes with different m6A modification patterns—each with its specific immunological profile. In contrast with patterns B or C, the pattern A modification had a higher number of invading immune cells and active immunological responses. The distinct immunological feature of each subtype also validated the feasibility of our classification method of the immunological phenotypes of the various m6A modulators. This immune subtyping technique may aid in the comprehension of fundamental processes of immune modulation, allowing for the development of more accurate treatment approaches. Thus, IA can be subtyped at the molecular or immunological level rather than merely at the phenotypic level. In recent research, this technique was utilized to identify two unique m6A modification patterns in low-grade gliomas, contributing to a better comprehension of the tumor microenvironment, which may aid in establishing more efficient immunotherapeutic treatments in the future (43). For IA, Chen et al. constructed co-expression networks using the WGCNA approach for ruptured and unruptured IA samples, examined gene modules, and screened genes regulating IA rupture, concluding that inflammatory and immunological responses may perform a crucial role in IA rupture (44). Interestingly, Song et al. demonstrated the imbalance of Th17/Treg in patients with IA, and the frequencies of Th17 cells were positively correlated with the severity of IA-induced spontaneous subarachnoid hemorrhage (45).

We identified m6A modulator-associated genes and modification patterns, as well as revealed their biological functions to explain the pathogenesis of IAs from the perspective of m6A modification. In addition, from the perspective of functional pathways, tight junctions were enriched in model A as opposed to model B. The Notch signaling pathway was remarkably enriched in model A as opposed to model C. The p53 signaling pathway was remarkably enriched in model C as opposed to model B. Finally, eight m6A methylation modification markers were identified: IGFBP2, IGFBP1, IGF2BP2, YTHDF3, ALKBH5, RBM15B, LRPPRC, and ELAVL1. Altogether, *IGFBP2, IGFBP1*, and *IGF2BP2* were overexpressed in IA samples, while *YTHDF3, ALKBH5, RBM15B*, and *LRPPRC* were overexpressed in normal samples. When comparing IA and normal samples, there were no differences in the expression levels of *ELAVL1*. *IGFBP2* is a protein-coding gene linked to diseases such as insulin-like growth factor I and malignant ovarian cysts. Its associated pathways include myofascial relaxation and contraction pathway and IGF-1 receptor signaling (46). The elevated expression level of this gene has been shown to accelerate the progression of numerous malignancies and may be used to anticipate the possibility of patient recovery (47). Recently, it has been shown that IGFBP2 induces selective polarization of pancreatic ductal adenocarcinoma macrophages *via* the STAT3 pathway, which leads to the macrophage-based immunosuppressive microenvironment in PDAC and thus promotes tumor progression (48). It has also been shown that increased levels of *IGFBP2* mRNA can anticipate an unfavorable survival status in patients with glioblastoma (49). In addition, in malignant melanoma, IGFBP2 modulates PD-L1 levels *via* the mechanism of activating the EGFR-STAT3 signaling pathway (50). On the other hand, *IGFBP1* is a protein-coding gene similar to *IGFBP2*. It has been shown that IGFBP1, a downstream protein of Jagged1, is related to the severity of coronary atherosclerosis among elderly patients, and aging-linked expression elevation in circulating IGFBP1 might be an adaptive response to counteract HCAEC aging *via* the Akt signaling pathway (51). Deng et al. showed that IGF2BP2 can bind to mRNA in an m6A-dependent manner and thus has the potential to become a new diagnostic and therapeutic target for patients with Alzheimer's disease (52). It has also been shown that SUMOylation of IGF2BP2 promotes angiogenic mimicry in gliomas by regulating the OIP5-AS1/miR-495-3p axis (53). In addition, YTHDF3, ALKBH5, RBM15B, LRPPRC, and ELAVL1 are all involved in m6A RNA methylation modifications (54). It has been shown that YTHDF3 acts as a negative modulator of antiviral immunity by promoting the translation of FOXO3 mRNA within equilibrium settings, thus providing deeper comprehension of the role of RNA-binding protein–RNA interaction networks in the maintenance of host antiviral immunological function and prevention of inflammatory responses in a balanced manner (55). Meanwhile, in reaction to positive single-stranded RNA virus infection, YTHDF3 acted as a positive modulator of antiviral JAK/STAT signaling, allowing Type I interferon (IFN)-mediated gene regulation programs to unfold in infected cells, suggesting that they are key response regulators in innate antiviral immune responses (56). Li et al. showed that ALKBH5 modulates the anti-PD-1 therapeutic response by regulating lactate in the tumor microenvironment and inhibiting immune cell accumulation (57). It was also shown that during HCV infection, the HCV non-structural 5A (NS5A) contributes to the suppression of the innate immune pathway by using LRPPRC to inhibit the ability of the mitochondrial antiviral signaling protein (MAVS) to regulate antiviral signaling (58). The above studies suggest that m6A indicators could be associated with immunological diseases and inflammatory responses, further demonstrating that m6A modulators could modulate immunological properties. Currently, studies on IA have focused on hemodynamics (59) and clinical therapeutic advances (60). In contrast, studies on the epigenetic modifications during IA are rare, with those on m6A RNA methylation modifications almost non-existent. We were the first to identify the function of m6A modulators in IA to explore their correlation with immunological features. The comprehensive findings of the present research show that m6A methylation modification introduces a unique research area in the study of the pathophysiology of IA.

There are certain limitations to the present research. First, we were unable to acquire additional clinical data for each patient, including age, Hunt-Hess grade, sex, treatment, as well as prognosis information, for longitudinal analysis. Therefore, we were unable to correlate m6A patterns, grading, and other clinical parameters for all samples. Second, even though we attempted to add as many samples as feasible in the GEO database that fit our criteria, the sample size remained constrained. Future research with larger sample sizes is needed. The specific m6A sites also should be illuminated by MeRIP-seq and MeRIP-qPCR to further identify the target genes of m6A regulators that were important for the intracranial aneurysms process. However, we are not qualified to obtain patient samples, which is why we have to use public data. In addition, some of the identified m6A regulators showed minor variation in expression between IA and normal samples; hence, more samples are needed for experimental validation. External datasets and tests, on the other hand, confirmed the excellent prediction accuracy of the m6A modulator gene characteristics that we identified. In addition, the correlation between the eight m6A markers we identified from the GEO datasets and IA. m6A methylation is thought to be critical in the onset and progression of IA, and we have shown compelling evidence to support this hypothesis.

In conclusion, we conducted a thorough investigation into the significance of m6A methylation among patients with IA, constructed an m6A regulator profile that distinguishes IA from normal tissue based on an eight-gene signature, and identified three distinct m6A isoforms according to 22 m6A modulators. The eight m6A regulators identified can be potential prognostic biomarkers for the treatment of IA. In addition, the three different m6A isoforms of IA showed remarkable differences in m6A regulator expression, the immunological microenvironment, and bio-functional pathways. These associations between immunological profiles and m6A isoforms provide insight into the development of novel targeted immunotherapies.

## Data availability statement

The datasets presented in this study can be found in online repositories. The names of the repository/repositories and accession number(s) can be found in the article/Supplementary material.

## Ethics statement

Ethical review and approval was not required for the study on human participants in accordance with the local legislation and institutional requirements. Written informed consent from the patients/participants or patients/participants' legal guardian/next of kin was not required to participate in this study in accordance with the national legislation and the institutional requirements.

## Author contributions

AM and MT: conceptualization, methodology, validation, investigation, supervision, software, visualization, writing the original draft, reviewing, and editing. XC, RS, KK, AA, DA, YA, RA, AK, ZW, and MA participated in the coordination of data acquisition and data analysis and reviewed the manuscript. All authors contributed to the article and approved the submitted version.

## Conflict of interest

The authors declare that the research was conducted in the absence of any commercial or financial relationships that could be construed as a potential conflict of interest.

## Publisher's note

All claims expressed in this article are solely those of the authors and do not necessarily represent those of their affiliated organizations, or those of the publisher, the editors and the reviewers. Any product that may be evaluated in this article, or claim that may be made by its manufacturer, is not guaranteed or endorsed by the publisher.

## Abbreviations

CDF: cumulative distribution function
DCA: decision curve analysis
DEGs: differentially expressed genes
GEO: Gene Expression Omnibus
GO: Gene Ontology
GSVA: gene set variation analysis
HLA: human leukocyte antigen
HNRNPC: HNRNP family of nuclear inhomogeneous proteins
IA: intracranial aneurysm
IFN: interferon
IGFBP2: insulin-like growth factor binding protein 2
KEGG: Kyoto Encyclopedia of Genes and Genomes
m1A: N1-methyladenosine
m5C: 5-methylcytosine
m6A: N6-methyladenosine
MAVS: mitochondrial antiviral signaling protein
NS5A: non-structural 5A
PPI: protein-protein interaction
RF: random forest
ROC: receiver operating characteristic
SIRT1: sirtuin 1
ssGSEA: single-sample gene set enrichment analysis
STAT3: signal transducer and activator of transcription 3
SVM: support vector machine
UIA: unruptured IA
WGCNA: weighted gene co-expression network analysis.
