# Supplementary figures and images for: m6A regulator–mediated RNA methylation modification patterns and immune microenvironment infiltration characterization in patients with intracranial aneurysms
Source: Front Neurol. 2022 Aug 5;13:889141. doi: 10.3389/fneur.2022.889141 (PMC9389407; doi:10.3389/fneur.2022.889141)

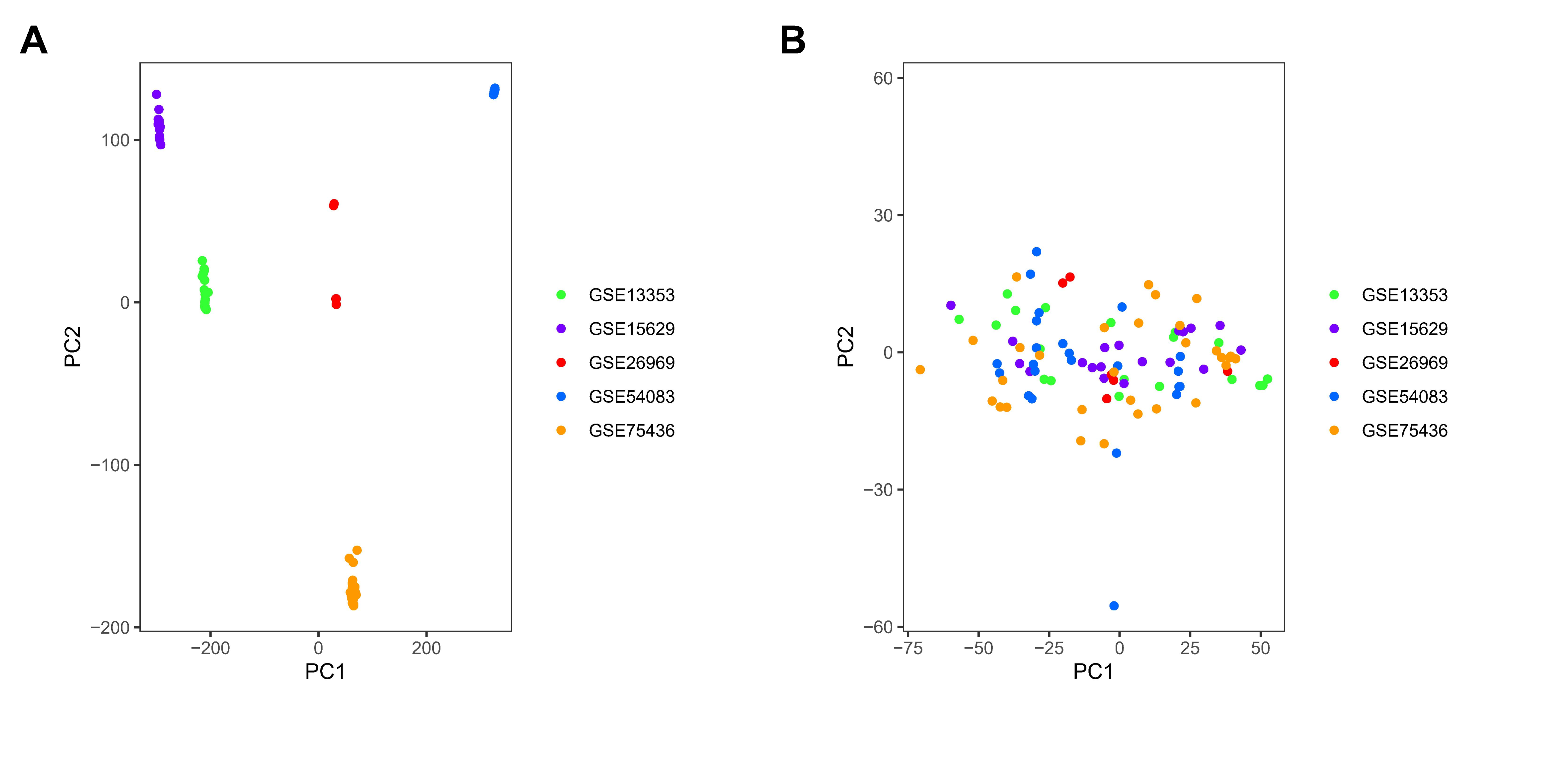

Supplement: Supplementary file 1 [file Image_1.TIFF]

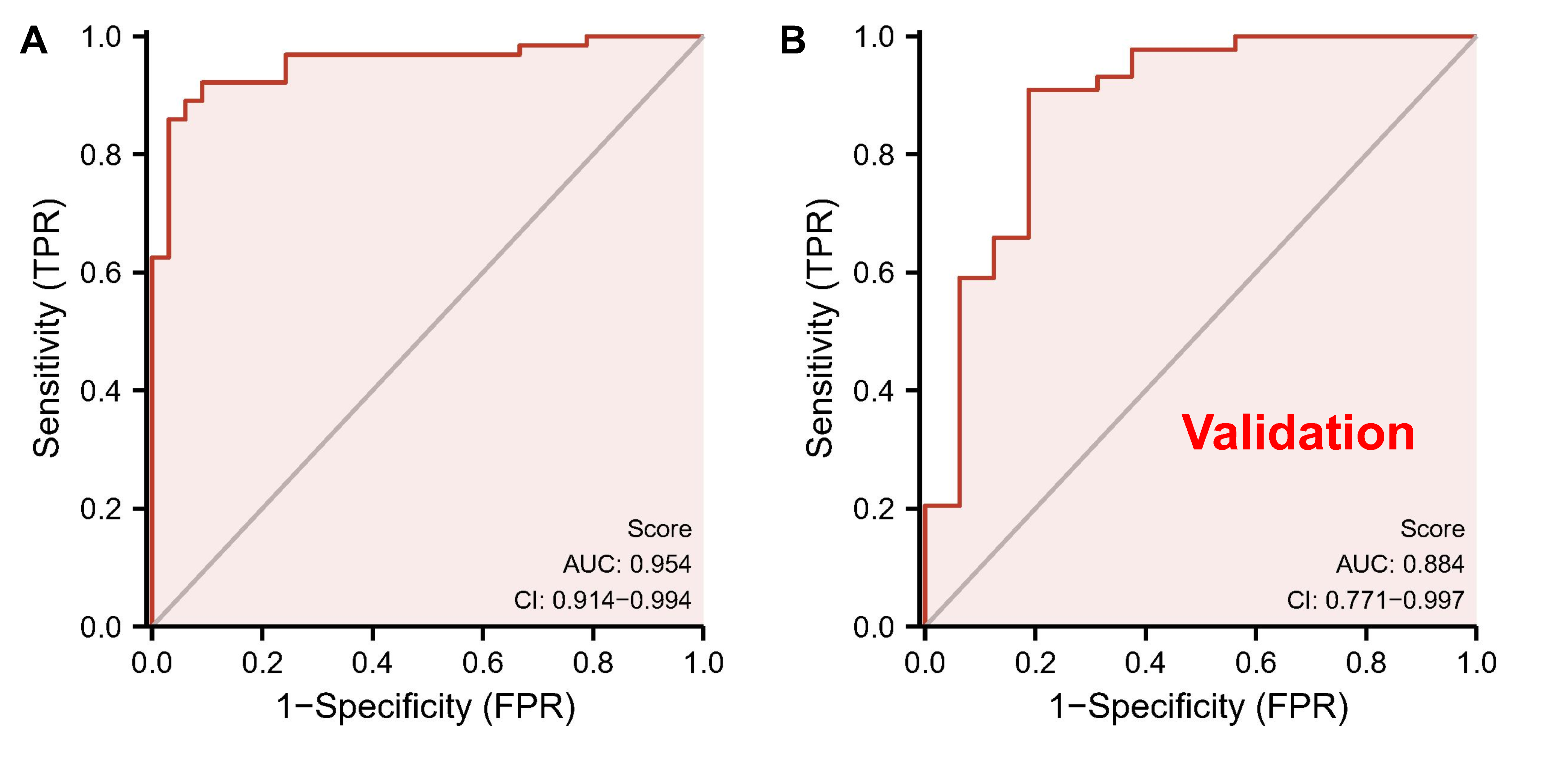

Supplement: Supplementary file 2 [file Image_2.tiff]

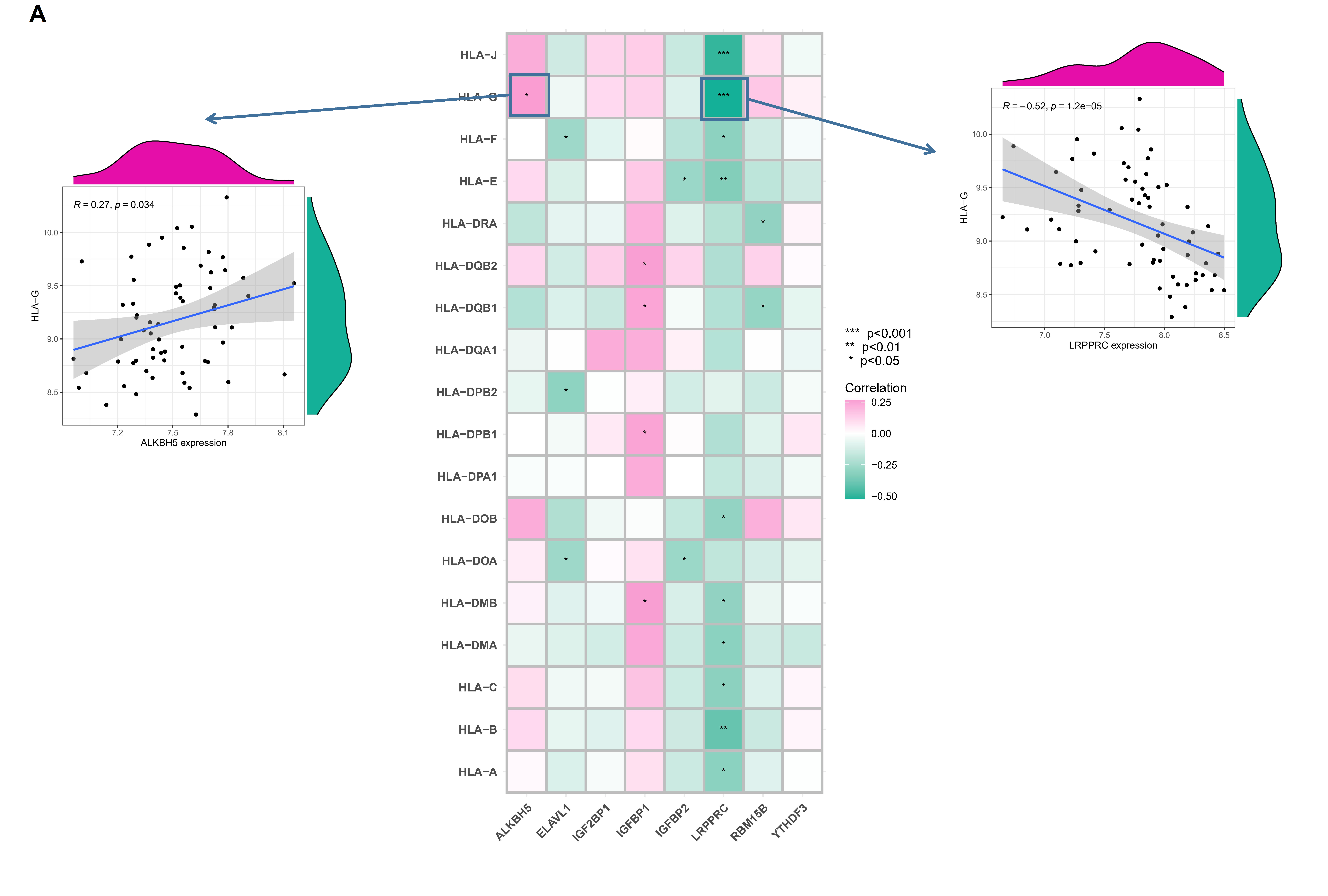

Supplement: Supplementary file 3 [file Image_3.tiff]
